# The Process of Thought; or, Electro-Biology
*The Process of Thought adapted to Words and Language, together with a description of the Relational and Differential Machines. By Alfred Smee, F.R.S. London: 1851.


**Published:** 1851-07-01

**Authors:** 


### Art. IV.—THE PROCESS OF THOUGHT; OR, ELECTRO-BIOLOGY.*

"Electro-Biology!" What is its meaning? Literally, the theory of life, explained by electricity, or the electrical theory of life, a new-fangled pseudo-science, ingeniously enough engrafted upon mesmerism. If the facts of mesmerism came before us, like Hamlet's Ghost, in a "questionable shape," those connected with electro-biology assume a still more dubious aspect, and certainly demand a much larger amount of heathen credulity. There are men at this moment "*starring*" about the country, performing feats of metaphysical jugglery, before crowds of well-dressed and intelligent people, who can no more explain what they see and hear, than they can the *leger-de-main* tricks of a French conjuror. There can be no doubt—and we give these comedy-acting philosophers the benefit of this admission—that one human being may, by performing certain antics, affect the senses of another. The annals of witchcraft abound with such cases. We can easily conceive the machinations of a reputed witch affecting the imagination and nervous system of certain persons—particularly females—in the darker ages; and we have the clearest judicial evidence on record, that a state analogous to catalepsy, accompanied by coma, convulsions, and sometimes profound physical insensibility, has been frequently so produced. The Lancashire Witches,† the Surrey Demoniac,‡ the Nuns of Loudun, exhibited all the symptoms now ascribed to mesmerism, and were subjected to the pin-thrusting test of sensibility, which is now so fashionable with mesmerists.§ In a treatise entitled 'Daimonomagia,' 1665, the "disease of witchcraft" is described as "a sickness that arises from strange and preternatural causes, and from diabolical power in the use of strange and ridiculous *ceremonies*, by witches or necromancers, afflicting with strange and unaccustomed symptoms, and, commonly, preternaturally violent, very seldom, or not at all, curable by natural remedies."‖

* The Process of Thought adapted to Words and Language, together with a description of the Relational and Differential Machines. By Alfred Smee, F.R.S. London: 1851.

† A briefe and true discourse, contayning the certayne possession and dispossession of seven persons in one family in Lancashire. By George More, Minister and Preacher of the Worde, now a prisoner at the Clinks, where he hath continued almost for two years. 1600.

‡ The Surey Demoniacke; or, an account of Satan's strange and dreadful actings in and about the body of Richard Dugdale, of Surrey, near Whalley, in Lancashire. London: 1697.

§ Véritable Relation des Justes Procédures observées au fait de la possession des Ursulines de Loudun et au procès de Grandier. Paris: 1634.

‖ Daimonomagia: a Small Treatise of Sicknesse and Disease from Witchcraft and Supernatural Causes. London: J. Dover, 1665.

There can be no difficulty in recognising the effects produced by what are called mesmeric manipulations; they have been exhibited, and are patent to the world; but we are not, in seeking an explanation of these effects, to have recourse to hypotheses which are purely imaginary. The mental faculties may, by a variety of tricks, be curiously enough disturbed. Thus, if a person be desired to keep a fixed and steady gaze on any inanimate object, the attention will become fatigued; and sleep, more or less profound, will ensue; and, in the intermediate state, between waking and sleeping, when the mind is wavering, and only half conscious of what is passing around, it may easily be imposed upon by any suggestion audibly communicated. Some years ago, the following curious experiment was performed at Manchester, before an audience of eight hundred persons:—Mr. Braid, who has written much upon what he terms 'Hypnology, or Nervous Sleep,' undertook to produce sleep by causing persons to gaze continuously at inanimate objects. Fourteen male adults, who had never tried the experiment before, stepped forward from among the audience to be experimented upon. He then desired some of them to keep a steady, fixed gaze upon the end of a cork, which he bound upon their head, so as to project over the eyes from the middle of the forehead. Each was then desired to look at the cork bound upon his own forehead, and concentrate his undivided attention on the act. Some also were desired to fix their sight and thoughts on other objects, as the gas apparatus in the room. They all commenced the process at the same time, and what was the result? Ten of the fourteen went to sleep, although the operator did not touch them until after their eyelids had closed involuntarily. None of them were able to open their eyes; some became cataleptic, others were insensible to the prick of a pin, and one or two forgot all that occurred. During these proceedings, three more of the audience threw themselves, of their own accord, into a state of profound sleep, by fixing their gaze and thoughts in the same way, upon other single points in the room.* There is nothing by any means so very marvellous in all this. Darwin, long ago, in his 'Zoonomia,' pointed out that, by fatiguing the attention, and thereby suspending volition, sleep would be produced. There is no doubt a great difference in the susceptibility of different people; as, indeed, was observed, in the more palmy days of witchcraft. "Aspect and contact do not always," according to Biernannus and Wierius, "bewitch; witches sometimes try to bewitch one, and cannot, and yet bewitch another of the same family."† But supposing, instead of the piece of cork, a disc of zinc, or a com-

* The Power of the Mind over the Body. By James Braid. London: Churchill. 1846.

† Daimonomagia, loc. cit.

mon brass button were substituted, and the same attention to it enjoined, would it be at all wonderful if the same effect were produced? Certainly not. Nay, we can easily understand that when the vision becomes confused, and the mental faculties bewildered, erroneous impressions and illusions, ocular and aural, may easily be suggested. "You cannot open your eyes," cries the electro-biological magician; his patient, half asleep and half awake, hears, believes, and immediately imagines his eyelids closed under a weight. In trying to open them, he makes no real muscular effort, the power of his volition is paralyzed, his eyelids remain closed, and the lecturer stands, with an air of intense pride and satisfaction, before his astonished audience.

The researches on magnetism by Baron von Reichenbach, recently published by Dr. William Gregory of Edinburgh, have excited considerable sensation; but, if fairly examined, his experiments will be found altogether inconclusive. The aim of the Baron is to establish the existence of a "new imponderable"—a something of magnetic origin akin to the supposed mesmeric fluid—affecting the powers and forces of all objects, animate and inanimate, organic and inorganic. There can be little doubt, that a magnet of a certain power drawn along the surface of the body, and in immediate contact with it, will produce certain physical sensation; and when at a little distance from the body sensitive persons may experience similar sensations in a modified degree. But the Baron von Reichenbach goes farther than this; he assures us that his patients, when in the dark, saw different coloured streams of light actually issue from the magnet.

The patients, we may premise, were principally sensitive females, and the Baron did not himself witness these extraordinary phenomena, neither was there any other corroborating testimony. The evidence, therefore, *in limine*, rests upon the individual report of each patient; but here, although we do not wish to be deficient in gallantry, we must say that the evidence of nervous and susceptible ladies, upon matters affecting their own impressions and their own senses, ought to be received with considerable caution. "It is an undoubted fact," observes Mr. Braid—and the profession will agree with him—"that with many individuals, especially with the highly nervous, imaginative, and abstractive, a strong direction of inward consciousness to any part of the body—especially if attended with the expectation or belief of something being about to happen, is quite sufficient to change"—or rather, we should say, to *modify*—"the physical action of that part, and produce such impressions from this cause alone, as Baron Reichenbach attributes to this new force."* Half a century ago, Dr. Haygarth, of Bath, by substituting wooden for the famous metallic tracters of Perkins, came

* Braid, loc. cit.

to the same conclusion. One of Baron Reichenbach's patients imagined that she saw the magnet giving out light, not only when open, but when closed. When open, she said the flames from the poles were about eight inches high, those at the joining of the plates of the magnet about a finger's breadth in length. These small flames appeared blue, the chief light being white below, yellow higher up, then red and green at the top—both poles seemed to give out similar appearances of light and flame; but, unfortunately, the same lady being again experimented upon, saw the streams of light issuing from the same magnet different both in size and colour. At the pole pointing to the north or negative end of the magnet, the flame was larger than at the opposite end; it was red instead of white below, and blue instead of yellow or red in the middle. There would clearly not have been this discrepancy had there been any physical reality in the alleged flames and colours. All doubt on the subject is, however, set at rest by the fact that in several of Mr. Braid's experiments, when he deceived the patient by pretending to use a magnet, all the abnormal sensations were produced by the mere imagination of the patient; as in Dr. Haygarth's case, the key of his portmanteau—when the patient imagined it to be a magnet—produced effects as singular as if it had been endowed with the new imponderable magnetic force discovered by the Baron's patients. Upon the very equivocal testimony of his nervous lady patients, the Baron conceives he has proved that this new imponderable force has the power of attracting the human body and adhering to it as steel to the magnet, and that it is a force in superaddition to the magnetic force in the magnet itself. Furthermore, that this imponderable is equally active in crystals, in which it exists pure and distinct from ordinary magnetism. He also believes that his patients saw, from the finger points of healthy men, fiery bundles of light streaming forth exactly as from the poles of magnets, and of crystals visible to the sensitive. He, moreover, alleges that where it is passive, it can be excited into activity by the sun's rays, by the moon's rays, by starlight, by heat, by chemical or mechanical action, and, finally, that this luminous or phosphorescent appearance, and certain other peculiar properties, may be discovered by the sensitive in almost every place, and from nearly every object, animate or inanimate. Assuredly this is the very poetry of science; it reminds us of a passage in the conversations of Goëthe with Eckerman and Soret. "We are all," said Goëthe, "groping among mysteries and wonders. Besides, one soul may have a decided influence on another, merely by means of its silent presence, of which I could relate many instances. It has often happened to me that when I have been walking with an acquaintance, and have had a living image in my mind, he has at once begun to speak of that very thing. I have also known a man who, without saying a word, could suddenly silence a party engaged in cheerful conversation by the mere power of his mind. Nay, he could also introduce a tone which would make everybody feel uncomfortable. We have all something of electrical and magnetic forces about us, and we put forth, like the magnet itself, an attractive or repulsive power, accordingly as we come in contact with something similar or dissimilar. It is possible, nay, even probable, that if a young girl were, without knowing it, to find herself in a dark chamber with a man who designed to murder her, she would have an uneasy sense of his unknown presence, and that an anguish would come over her which would drive her from the room to the rest of the household."* There is something certainly droll in the notion of our being human magnets; it is even alleged that sensitive patients should lie with their heads towards the north, and that they will suffer great discomfort if they do not, when nestled in their bed, place the position of their body in proper relation to the magnetic meridian. Hence, a young lady, writing to Mr. Braid, expresses herself in the following lively terms:—"You will, no doubt, be gratified to learn, that in common with the whole human race, you are a magnet, with this peculiarity only, that if you were suspended by the middle you would point from east to west instead of from north to south—with which interesting fact I leave you to your meditations."†

The discoveries which have been made by Ampère, Faraday, and others, in electro-magnetism, and the recent observations of Matteuci on the evolution of electricity during muscular contraction, suggest not only an analogy between the imponderable agents—light, heat, electricity, and magnetism—but the probability that they are modifications only of the same subtle principle. Many physiologists, indeed, entertain the opinion that the nervous fluid itself has some affinity with, or is perhaps identical with, electricity. This view appears to have suggested the term "Electro-biology," which—we quote, and beg attention to Mr. Smee's definition—"signifies neither more nor less than the relation of electricity to the vital functions." Unhappily for the interests of science, there is a disposition in the human mind to go beyond the actual limits of what is really known, in order to speculate upon things which are unknown, and no sooner is any one discovery supposed to be made, than a host of inconsequential theories immediately spring out, or are based upon it. This is eminently the case with electro-biology, which, under the mask of science, puts forth assumptions, which are ludicrously untenable, and promulgates doctrines which are eversive of the immateriality and independent existence of the

* Conversations of Goëthe with Eckermann and Soret. Translated from the German by John Oxenford. 2 vols. London: Smith and Elder. 1850. Vol. I. Page 150.

† Braid, op. cit. p. 26.

human mind. It is materialism in its most repulsive form, pretending, in its presumptuous speculation, to explain the mystery and origin of life. These mistaken philosophers believe that they can positively, by the action of a galvanic battery, create life; that they can call into existence a multitude of insects, of which the *acarus Crossii* is a notable example; and, having allowed them their premises, they then proceed to show how every fundamental organic globule is endowed with a principle of self-evolution, and how, by an easy process of gradual development, one species of animal passes into another species of animal until reptiles become fish—fish, birds—birds, monkeys—and monkeys, men. Happily, however, for the stability of the insect-world, the more enlarged observations of Ehrenberg completely overthrow the vaunted experiments of Mr. Cross, who can no more, with the aid of his galvanic battery, give life to an acarus than to a cockchafer, a tadpole, or a pullet's egg. In the "Elements of Electro-Biology," by Mr. Alfred Smee, we find that he far outrivals—at least in his attempts—the insect-maker, Mr. Cross. He gives very clear instructions how to make an artificial electric fish. "Catch your hare first," says Mrs. Rundell; but it is not necessary to catch, you must, *secundem artem,* in electro-biology, *make* your fish. "This artificial electrical fish," says our electro-biological philosopher, "is made by taking an ordinary solution of ferrocyanate of potash contained in a glass-vessel. In this glass-vessel a porous cell, with a similar solution, is introduced. Now, if a series of these cells be taken and connected together by platinum wires, so arranged that the inside of the porous cell of one vessel be connected with the exterior of the second by a platinum wire, no action will be indicated by the galvanometer. If, however, a current of voltaic electricity be now passed through each cell, from the porous tube to the exterior, one department, or the hydrogen side, will become alkaline, and the salt will retain its chemical character; the other cell will become acid, and be converted into the red prussiate."* In designing this "abundantly complex" mechanism, we cannot conceive any feasible source from which the principle of vitality could, after all, be eliminated; the projectors and builders of the Tower of Babel were as far from reaching Heaven as these philosophers are from approaching the solution of the mystery of life. Could "Man, proud man —dressed in a little brief authority," thus mimic the works of his own Maker, and set a-going an infinite transmutation of species, beginning with a fundamental cell, an organic globule, what anomalies and

* Elements of Electro-Biology, or, the Voltaic Mechanism of Man; of Electro-Pathology, especially of the Nervous System; and of Electro-Therapeutics. By Alfred Smee, F.R.S. Longman: 1850. p. 55.

wonders would not be ever-and-anon starting into existence under our own immediate observation.

From the same electro-biological authority we learn, "that the ganglia of the sympathetic nerves are rudimentary brains, which might be evolved in structure to such an extent as to render us cognizant of minute changes taking place in our own bodies, but which for many hundred filaments only send a single impression to the noemic batteries in the brain."* Alas! for the safety and integrity of our own great solar plexus, and the cranial protection of our "central" cerebral battery. This "gradual development" and "self-evolving principle" in the inorganic and organic world; this transmutation of one specific being into another, might suggest considerable uneasiness about our own personal identity. "Under such celestial treatment," says a severe but acute critic, "we might live to see a grizzly dowager, a wheezing bachelor, and a withered maid, sitting down to a quiet game at whist, with a new-fashioned dummy in the form of a solemn poodle, while a lively spitz, or fawning spaniel, is raised on its hind quarters at the end of the sofa, and teaching the knight's move to the younger ladies of the household."†

Wishing, notwithstanding all this, to give the principles of electro-biology a fair hearing, we were curious to ascertain what light it could possibly throw upon the constitution of the human mind, and finding the book which suggested this article, "*The Process of Thought adapted to Words and Language*," floating on the surface of literature, we determined to analyze its contents; and we regret to say, our worst apprehensions are more than verified; paragraph upon paragraph numerically succeeding each other, as if each should be accepted as a canon in philosophy, will be found to contain the most common-place description of truisms wrapt up in the most solemn and pompous phraseology, masking at the same time the most daring materialistic speculations. We shall allow the author to state his own aphorisms in his own language; his style appears to us both obscure and often unintelligible; but metaphysics has been wittily enough defined, "*l'art de s'égarer avec méthode.* As the paragraphs are numbered, so do we take them. The first propounds the following dogma, which is delivered as abruptly as if it were articulated by an oracle. "(1.) The perfection of the operation of the brain by which man performs the noblest attributes of his nature, can no more be enhanced by a know-

* Elements of Electro-Biology, or the Voltaic Mechanism of Man; of Electro-Pathology, especially of the Nervous System; and of Electro-Therapeutics. By Alfred Smee, F.R.S. Longman: 1850. Page 79.

† Edinburgh Review: Article on the "Natural Vestiges of Creation." Vol. lxxxii. page 7.

ledge of its organization, than the working of a steam engine could be improved if it could be made to know the mechanism by which it obtained its desired result," p. 1. Here we must emphatically throw in an interlocutor. We protest, *in limine*, against the assumption that the noblest attributes of man are to be ascribed to the mere *functional* operations of his brain; we regard the mind, although in its manifestations influenced by matter, as something higher than the result of mere cerebral organization; surely, if man be ennobled by his moral attributes, or exalted by his intellectual faculties, it is to the mind that we must refer this ascendancy. The clumsy illustration of the steam engine we repudiate; if a steam-engine were capable of reflecting upon its own springs, levers, pumps, pistons, valves, and mill-wheels, we do not see why it might not improve upon its own working, just as man, by reflection and self-control, may improve his own moral and intellectual nature. "(2.) Electro-biology teaches, that *man* receives *impressions* from the external world through the medium of his organs of sensation, transmits those impressions to the brain, and there registers them in such a manner as to render the sensorium one vast mechanism, in which everything that has been heard or seen, or felt, or smelled, or touched, has produced an effect which modifies the action of any impression which may be subsequently received," p. 2. Now, when the electro-biological philosopher teaches us that "*man* receives impressions from without, and that man transmits these impressions to the brain, in order to register them in the sensorium," what does he mean? it is the *mind* of man, we apprehend, which *receives* these impressions, and *performs* these operations. But, no! the electro-biologist does not recognise the existence of a thinking, intelligent principle, apart from cerebral organization. He adopts an exploded theory, which Reid long ago censured in these terms: "Some philosophers among the ancients, as well as among the moderns, imagined that man is nothing but a piece of matter so curiously organized, that the impressions of external objects produce in it sensation, perception, remembrance, and all the other operations we are conscious of. This foolish opinion could only take its rise from observing the constant connexion which the Author of Nature hath established between certain impressions made upon our senses, and our perception of the objects by which the impression is made; from which they weakly infer, that those impressions were the proper and efficient causes of the corresponding perception. But no reasoning is more fallacious than this, that because two things are always conjoined, therefore one must be the cause of the other. Day and night have been joined in constant succession since the beginning of the world; but who is so foolish as to conclude from this that day is the cause of night, or night of the following day? *There is indeed nothing more ridiculous than to imagine that any motion or modification of matter should produce thought.* If one should tell of a telescope so exactly made as to have the power of seeing; of a whispering-gallery that had the power of hearing; of a cabinet so nicely framed as to have the power of memory; or of a machine so delicate as to feel pain when it was touched,—such absurdities are so shocking to common sense, that they would not find belief even among savages; *yet it is the same absurdity to think that the impressions of external objects upon the machine of our bodies can be the real efficient cause of thought and perception.** We must, however, give our philosopher credit for the amusing notion of the sensorium being "one vast mechanism," in which man registers everything he has "heard, seen, felt, smelt, or touched;" the idea was, doubtless, suggested by the Crystal Palace, and completely eclipses the cave of Plato, and the dark-chamber illustration of Locke.

Electro-biology next teaches (3) "that every idea or action on the brain is ultimately resolvable into an action on a certain combination of nervous fibres which is definite and determinable; and, regarding the sum total of the nervous fibres, is a positive result over a certain portion only, which has a distinct and clearly defined limit. Thus, if we take ten nervous fibrils and call them A, B, C, D, E, F, G, H, I, J, and suppose an action to have occurred on D, E, F, the combination, excited to action, will give rise to an idea which would depend upon their positive excitement, and the positive character of the idea would be limited to that combination. Instead of using the letters D, E, F, I may illustrate the proposition by assuming the fore-finger to represent those letters, when it would be apparent that if that finger was placed in hot water, the idea of that particular action of the hot water would be confined to the nerves supplying that part."—p. 2. Here, again, our philosopher appears to have sadly bewildered himself. What does he mean by using synonymously the words "*every idea*" and "*action on the brain?*" Are we to understand that every idea is an action *on* the brain? or every action of the brain in itself an idea? Then, again, resolving these into an imaginary combination of nervous fibres represented by the first ten letters of the alphabet, and shifting these about like a child's toy, in order to show how these fibres may be excited to different action, is as absurd a piece of conjuring as can possibly be conceived. The theory of Hartley was at all events ingenious, but even he, with all his vibrations and vibrunaili, failed to account for the operations of thought. "Let us," says Blakey, "admit all that the

* Reid on the Intellectual Powers. Essay II. Chap. iv. p. 253. Sir William Hamilton's Edition of Reid's collected Works. Edinburgh: 1846. This valuable work ought to be in the hands of every student of psychology.

doctor demands—what is the result? Suppose that the nerves were to vibrate, and we were to find thinking or mental perception followed, this would not enable us to account for any intellectual operations; for we should just be as far from the comprehension as before. If the brain and the whole nervous system were laid bare, as we display the wires in a pianoforte, and we could see all the various vibrations which are effected by the operation of external objects upon the senses; still we should be as far removed from a knowledge of the thinking principle as we were before such wonderful mechanism was exhibited to our view. We might register every movement; we might observe, with great accuracy and minuteness, various connexions between particular medullary motions and particular trains of thought; and yet there would not one single ray of intelligence fall upon 'the sightless eyeball of the mind,' as to the nature or principles of its operations. This is evidently beyond the reach of theory."* Unfortunately, also, for the fibril theory of electro-biology, the most recent physiological and pathological researches lead us to believe that the vesicular, and not the fibrous—the grey, not the white—matter of the brain, is concerned in mental operations. We have not time to amuse ourselves with dilating upon the absurdity of the alphabetical illustration; but we cannot help observing, that if we dipped our finger in hot water, the *idea* of the pain would not, we conceive, be confined to the scalded finger—would it not be carried up and registered in that "vast mechanism," the sensorium? The sense of touch, we apprehend, is not exactly confined to the tips of the fingers, although a blind child may be taught to read with his fingers, and, as Sidney Smith wittily observes, learn to "*feel* his way through Homer and Virgil."

The fundamental principles of electro-biology, which its professor conceives will throw light upon the "process of thought" and illustrate the meaning of words and construction of language, are laid down in the following terms. "(8.) In the preceding chapter," he observes—and this is the summary of its logic,—"I have stated that external objects act upon the organs of sensation (!) that that action is transmitted to the sensorium (!!) and that it is probably registered in a certain combination of nervous elements, to appear again on subsequent occasions, constituting memory (!!!)" p. 5. A system of philosophy, based upon so crazy a foundation, can hardly be expected to elucidate the meaning of words and the construction of language; nevertheless, we are carried back to the grammar of our vernacular tongue, and confess, that under this electro-biological tuition the use of words (as our philosopher states) "becomes a very complex phenomenon," p. 6. But what are

* History of the Philosophy of the Human Mind. By Robert Blakey. 4 vols. London: 1848. Vol. iii. p. 281.

words? The simplest operation of our minds must be expressed by certain sounds; and by putting together a given number of letters, words are formed, which symbolize and are associated with certain visible objects, or certain ideas, passing through the mind. "Words," says that great authority, John Murray (see his school grammar), "are articulate sounds, formed by the organs of speech, and used by common consent as signs of our ideas." We confess we remain contented with this definition, which we learned in our school-boy days; but electro-biologists must fain desire more; "words," according to them, are used to "represent various images impressed on the brain," p. 6,—these are ideas which we presume the brain reflects as a mirror would objects around it. "The letters of the alphabet are used to designate certain combinations of nervous fibres;" so that one of Dr. Johnson's rounded sentences must express a tolerable amount of intertwisting among these brainular fibres. What, then, is a substantive? Electro-biology teaches us, that "a substantive is a part of speech given to the action on a combination of nervous elements, which are affected in common by a large class of objects, and is therefore in itself a very general term," p. 7. This is just the definition we should have supposed that great philosopher Crabbe putting into the mouth of Martinus Scriblerus. How much better would it be for such philosophers as these to go back to their Eton Latin grammar, where, without puzzling themselves about their nervous fibres, they might learn *simpliciter*, that "a noun substantive declares its own meaning, and requires not another word to be joined with it to show its signification—as *homo*, a man; *angelus*, an angel; *liber*, a book." So also with adjectives; we had always in our simplicity believed that an adjective was added to a substantive, to express its quality—or, as our Eton preceptor taught us, "a noun adjective always requires to be joined with a substantive, of which it shows the nature or quality; as, *bonus puer*, a good boy; *malus puer*, a naughty boy." The etymology of electro-biology, however, is far more recondite—it teaches us that an adjective is used to denote some further combination of cerebral actions. "When we use a word adjectively, and couple it to a noun, the adjective implies that only a portion of the actions of the brain, which led to the idea from whence the word is derived, are coupled with the noun; hence, as the amount varies, we have various degrees of the word used adjectively; as, good—better—best." (18.) p. 9. As the amount of cerebral action becomes augmented, we have the positive eliciting the comparative, and the comparative the superlative degree; truly, this is charming philosophy! The clown in the forest, in "As you Like it," giving Audrey such a lecture as this, would draw down from the audience shouts of laughter. But we cannot afford room to entertain ourselves with the electro-biological definition of all the parts of speech; suffice it, therefore, that we conclude with the account given of the meaning of a verb. "A verb" (says our philosopher) "we may electro-biologically define to be a word used to signify the changes on the sensorium of the respective portions of one image, and their relation to those of other images." (24.) p. 11. Shade of Murray defend us! But what follows? "To explain this definition, it is important to remember that the brain is one large organ, on which a series of impressions are being continually made, both from the action of external agents upon the organs of sensation, as well as from the changes going on in our own frame." (25.) p. 11.

The adaptation of electro-biology to the etymology of words and construction of sentences is, however, best illustrated in the following paragraph. "In many cases verbs have relations to two substantives, as, 'John killed Thomas.' In this expression we understand that at some time past the act of killing was done by John on Thomas; the first individual performed certain actions which caused a second set of actions to supervene on Thomas. The verb here modifies the ideas which we derive of both nouns; and the sentence gives us the idea of at least three different states—

"First. John and Thomas, both alive.

"Second. John in action, Thomas being acted upon.

"Thirdly. John alive, Thomas dead.

"These series of changes or sequences stand in relation as cause to effect, and, in language, may be rendered that John caused the death of Thomas." (45.) p. 21.

Let us take another example:—"'John and Thomas killed William.' Let J stand for John, T for Thomas, C for casualty, D for death, E for effect, W for William, P for the past; which, electro-biologically, would point to different distinct ideas, having mutual relations, thus:

JTC WEDP.

In the first place, John and Thomas underwent certain changes, in consequence of which, in the second place, William underwent certain changes to death, the whole happening at some time past."—(49.) p. 50.

So much for the adaptation of electro-biology to the use of words and the construction of sentences;—a set of such philosophers as these we can only conceive giving lectures in the flying island of Laputa;—nay, we prefer, infinitely, the philological theory of Will Wizard, whose philosophical turn of mind led him, in Salmagundi, to make this profound observation,—"*Words are but breath—breath is but air—and air, put in motion, is nothing but wind.*" Here the relation between cause and effect—antecedent and consequent—must be manifest, to the satisfaction of every electro-biologist.

